# A systematic review of the Nexus between campus green spaces and mental well-being in mainland China and Hong Kong

**DOI:** 10.3389/fpsyg.2026.1846925

**Published:** 2026-06-19

**Authors:** Jueshan Liu, Yumei Chen, Anthony Orji

**Affiliations:** 1 Sichuan Vocational College of Health and Rehabilitation, Zigong, Sichuan, China; 2 Department of Economics, University of Nigeria, Nsukka, Enugu, Nigeria

**Keywords:** campus green spaces, environmental health, green infrastructure, mental well-being, psychological well-being, student mental health, systematic review

## Abstract

**Objective:**

This systematic review examines the association between campus green spaces and mental well-being among university students, with a specific focus on evidence from mainland China and Hong Kong. It synthesizes findings on how different measures of greenness relate to mental health outcomes, including stress, anxiety, depression, life satisfaction, and psychological well-being.

**Methods:**

The systematic search was conducted in compliance with Preferred Reporting Items for Systematic reviews and Meta-Analyses (PRISMA) 2020 and the Preferred Reporting Items for Systematic reviews and Meta-Analyses literature search extension (PRISMA-S) extension for reporting search strategies. Five databases (Web of Science, PubMed/MEDLINE, CNKI, CrossRef, and OpenAlex) were searched. Studies were screened against predetermined eligibility criteria, and data were extracted and synthesized using a structured narrative approach due to methodological heterogeneity.

**Results:**

A total of 988 records were identified, of which 22 studies were selected after initial screening and full-text assessment. Most studies have shown that people who spent time in campus green spaces experienced improved mental health results, including lower levels of stress, anxiety, and depressive symptoms and greater life satisfaction. Studies reported more reliable results using subjective greenness assessments than with objective measurements such as NDVI. Identified pathways included physical activity, social interaction, and place attachment, which affected the relationship between the two variables.

**Conclusion:**

The result indicates that exposure to campus green spaces is associated with better mental health among university students in mainland China and Hong Kong. While the evidence base is methodologically diverse, findings demonstrate that campus green infrastructure, as part of mental health promotion techniques, is a cost-effective and scalable measure. This review provides a geographically focused synthesis of evidence from mainland China and Hong Kong, contributing to the understanding of how campus green spaces relate to student mental well-being in high-density urban contexts.

## 1 Introduction

Mental well-being is a multidimensional construct encompassing both hedonic components (e.g., happiness, life satisfaction) and negative psychological states (e.g., stress, anxiety, depression). The increased rate of urbanization, the increased pressure on academic achievements, and the modified patterns in the socio-cultural expectations altogether have augmented the mental health problems of university pupils worldwide. It has been estimated in all parts of the globe that about 21 percent of first-year students report having experienced depressive symptoms (Wong et al., 2006), and about 38% have reported experiencing hopelessness (American University Health Association, 2006).

This is especially severe in mainland China and Hong Kong, where competitive academic cultures, high-stress examination cultures, and transitional life strain interact and exert pressure on university populations (Zhai et al., 2018b; Sze et al., 2021). Additional comparative studies imply that Chinese pupils tend to be more stressed and psychologically ill than their Western colleagues (Wang et al., 2017), which contributes to the urgency of having convenient mental health services that are culturally relevant.

Green spaces, defined as natural or semi-natural environments such as parks, gardens, and vegetated campus landscapes, have been increasingly recognized as important environmental determinants of mental health (Mensah et al., 2016) have increasingly been accepted as protective environmental determinants of mental health. The relationship between the two elements operates through two fundamental theoretical frameworks. Attention Restoration Theory (Kaplan, 1995) and Stress Reduction Theory (Ulrich, 1983) suggest that exposure to natural environments promotes recovery from cognitive fatigue and reduces physiological stress through affective and autonomic pathways (Wendelboe-Nelson et al., 2019; Zhang et al., 2020). However, important gaps remain regarding the optimal dosage, quality, and accessibility of green space exposure. Furthermore, the mechanisms through which these environments influence mental well-being are not yet fully understood. Proposed pathways include physical activity, social cohesion, and perceptual restoration (Chen K. et al., 2021). Emerging empirical studies have consistently demonstrated a positive association between campus green environments and students’ mental health outcomes. Increased green cover on campuses has been associated with reduced stress, improved emotional conditions, and enhanced restorative experiences (Lu et al., 2022; Bai X. et al., 2024). However, three key limitations constrain the generalizability of these findings. First, most studies are cross-sectional and rely on self-reported measures, which limit causal inference and introduce potential bias (Gao J. and McLellan, 2018). Second, variability in the operationalization of green space, including objective measures (e.g., NDVI) and subjective assessments (e.g., perceived accessibility and quality), limits comparability across studies (Suppakittpaisarn et al., 2017). Third, mediating mechanisms such as physical activity, place attachment, and social cohesion remain underexplored in East Asian university contexts (Koay and Dillon, 2020; Lai and Ma, 2016a).

Also, the socio-environmental context in mainland China and Hong Kong may mediate the effects of green spaces on students’ mental health. The dense urbanization, enclosed or remote campus developments, collectivist cultural orientations, and strict academic performance standards can magnify the impact of green space compared with Western settings (Xue et al., 2016). Other conditions, including congested dormitories, limited access to campuses, or mobility restrictions during pandemics, have also increased the salience of on-campus green infrastructure to Chinese students (Bai Y. et al., 2024). Furthermore, nature-relatedness, stress coping, and help-seeking behavior, as well as cultural differences, can further moderate the relationship between green space and well-being (Putra et al., 2020).

Mental well-being is a multidimensional construct that encompasses both hedonic and eudaimonic dimensions of psychological functioning (Diener, 1984; Ryff, 1989). Hedonic well-being refers to subjective experiences such as happiness, life satisfaction, and emotional balance, whereas eudaimonic well-being reflects psychological functioning, purpose, and self-realization. Furthermore, mental well-being research often includes negative psychological states such as stress, anxiety, and depression, which represent key indicators of psychological distress (Keyes, 2002). Mental well-being is used as an umbrella construct encompassing both positive psychological functioning and negative mental health indicators.

In this review, an integrative conceptual framework was adopted that incorporates both positive (hedonic and eudaimonic) and negative dimensions of mental well-being to provide comprehensive coverage consistent with contemporary psychological approaches.

### 1.1 Study objectives

This systematic review aimed to answer the research question using the population, intervention, comparator, and outcomes (PICO) framework: Participants (university students in mainland China and Hong Kong), Intervention (exposure to campus green spaces), Comparison (differences in levels or absence of green space exposure), and Outcomes (mental well-being indicators including stress, anxiety, depression, and life satisfaction).

Accordingly, the main aim of this review was to synthesize the empirical evidence on the relationship between exposure to campus green space and mental well-being among university students in mainland China and Hong Kong.

Specifically, the review aims to: (1) assess the nature of green space measures (objective or subjective); (2) assess the consistency and direction of the relationships between green space exposure and mental well-being outcomes; and (3) determine the key mediating and moderating variables involved in the relationships.

## 2 Materials and methods

### 2.1 Study design

This systematic review was conducted and reported in accordance with the PRISMA 2020 statement (Page et al., 2021) and adhered specifically to the PRISMA-S extension for reporting the literature search strategy. To provide transparency, reproducibility, and methodological rigor across all steps, the literature search, study selection, data extraction, and synthesis were included in the review.

The review was conducted in a structured protocol that included (1) the process of identifying the relevant studies via the extensive use of databases, (2) the process of screening records based on the preset inclusion criteria, (3) the full-text analysis of the inclusion, (4) the systematic and (5) the narrative process of synthesizing the findings since the studies included become methodologically heterogeneous.

### 2.2 Eligibility criteria

Only studies conducted within mainland China and Hong Kong were eligible for inclusion. Studies conducted in other geographic contexts were excluded during the full-text screening stage.

For this review, mental well-being was operationally defined as a multidimensional construct encompassing both negative and positive dimensions of psychological functioning.

Negative mental health indicators included stress, anxiety, depression, and psychological distress, quantified by feasible measures like the Perceived Stress Scale (PSS), Generalized.

The variables are the anxiety disorder scale (GAD-7), patient health questionnaire (PHQ-9), and depression anxiety.

Positive mental well-being indicators included constructs such as life satisfaction, emotional well-being, happiness, and psychological restoration.

Research was included if it assessed at least one of these predefined mental health outcomes using appropriate measures or indicators.

#### 2.2.1 Inclusion criteria

The following criteria were included in this study:

- Population: University or university students make up the targeted group for the study.
- Exposure: The study defined campus green space exposure through the assessment of natural and vegetated areas that students encounter throughout university grounds. The study included both objective assessment methods, which used NDVI and GIS-based greenness indices and spatial buffers, and subjective assessment methods, which included perceived greenness, accessibility, and quality and use of green spaces.
- The study excluded all research that investigated environmental effects that did not include vegetative elements through their research.
- Outcomes: The study assessed mental well-being through validated instruments, which measured a multidimensional psychological state that included both negative psychological states and positive psychological functioning.
- Study design: Empirical quantitative, qualitative, and mixed-methods studies, including cross-sectional, longitudinal, and experimental or quasi-experimental designs.
- Geographic scope: Studies conducted in mainland China or Hong Kong.
- Publication characteristics: Peer-reviewed journal articles published in English.

#### 2.2.2 Exclusion criteria

The following criteria were excluded from the study:

- Did not involve university or university student populations.
- Did not examine campus-based green space exposure.
- Did not assess mental well-being outcomes as defined in this review.
- Were conducted outside mainland China or Hong Kong.
- Were non-empirical publications (e.g., reviews, commentaries, editorials, protocols).
- Did not provide sufficient methodological detail or outcome data for analysis.

These criteria were maintained across the study during the screening and full-text review stages.

### 2.3 Information sources

A comprehensive literature search was conducted across five electronic databases: Web of Science, PubMed/MEDLINE, China National Knowledge Infrastructure (CNKI), CrossRef, and OpenAlex.

Scopus was initially considered but could not be accessed; therefore, it was excluded from the final search. To ensure transparency, only databases that were fully searched have been reported.

The number of records retrieved from each database was documented to ensure transparency and reproducibility.

Furthermore, backward reference searching was performed on all included studies to identify any additional relevant articles not captured in the primary database searches. The selection of databases was intended to ensure comprehensive coverage of both international and Chinese-language literature relevant to the study. Although databases indexing Chinese-language literature (e.g., CNKI) were included to enhance search coverage, only English-language studies were included due to resource and translation constraints. This may have led to the exclusion of relevant studies published in Chinese, introducing potential language bias.

Duplicate records were identified and removed prior to screening using Zotero. The final literature search was conducted on 15 March 2026, and all databases were searched on the same day to ensure consistency.

### 2.4 Search strategy

A search strategy was developed by combining controlled vocabulary (e.g., MeSH terms) and free-text keywords to identify studies that investigated the relationship between exposure to campus green space and mental well-being among university students.

The search was organized around three major concept groups: (1) green space exposure, (2) mental well-being outcomes, and (3) the population of university students. Terms within each concept were combined using the Boolean operator, OR, and the three groups were then joined with the operator AND. The mental well-being category included both positive psychological constructs (e.g., life satisfaction, happiness, emotional well-being) and negative psychological conditions (e.g., stress, anxiety, depression, psychological distress), consistent with the operational definition used in this review.

Regulated vocabulary terms were used in databases that facilitate indexed subject headings, such as PubMed and Scopus, which were augmented with free-text keywords to increase sensitivity and specificity. In databases that lack command-of-vocabulary indexing (e.g., Google Scholar and CNKI), similar free-text search terms were employed.

An example of the full search strategy used in PubMed is provided below:

(“green space” OR greenness OR “green infrastructure” OR vegetation OR NDVI OR “normalized difference vegetation index” OR “urban green” OR “campus green space”)

AND

(“mental well-being” OR “psychological well-being” OR “life satisfaction” OR happiness OR stress OR anxiety OR depression OR “psychological distress”)

AND

(university OR university OR campus OR “higher education” OR students)

Each database had its own adopted search strategy based on its indexing systems and syntax. Search terms had also been limited (where necessary) to the title and abstract fields to enhance precision.

Result filters were also used to narrow the results to English-language peer-reviewed journal articles. No date limitations were imposed during the search process, but articles published between 2000 and 2024 were included in the present research. Notably, all studies included in the present research were published between 2020 and 2024, indicating the relatively recent development of the field.

Furthermore, a secondary search was performed using Google Scholar to identify relevant studies not covered by the selected databases that were initially searched.

The original search strategy was designed to be broad to maximize sensitivity and cover the limited literature on the topic. This resulted in a large percentage of records being filtered out during the title and abstract screening phase, consistent with the standard systematic review methodology.

All databases are accompanied by full search strategies in Supplementary material 1, thereby enhancing transparency and reproducibility.

### 2.5 Study selection

The entire selection process, including the number of records identified, screened, excluded, and included, is presented in the PRISMA 2020 flow diagram.

All detected records were converted into a reference management system, and redundant records were eliminated before screening. The selection of the studies was performed in three phases, namely: (1) screening the title, (2) screening the abstract, and (3) appraising the full text.

All records were independently screened by two reviewers using titles and abstracts to focus on potentially eligible studies. The potentially relevant articles were subsequently used to retrieve full texts that were evaluated for compliance with the predefined inclusion and exclusion criteria.

All records were independently screened by two reviewers at both the title/abstract and full-text stages. Discrepancies between reviewers were documented using a structured screening log maintained in the reference management system.

A total of 27 disagreements were identified during the title and abstract screening stage, and 11 disagreements during full-text assessment. Initial discrepancies were discussed between the two reviewers to reach a consensus. In cases where agreement could not be achieved (*n* = 6), a third reviewer acted as an adjudicator and made the final decision.

The consensus process followed a structured sequence: (a) independent screening by two reviewers; (b) comparison of screening decisions and identification of discrepancies; (c) discussion between reviewers to resolve disagreements; and (d) adjudication by a third reviewer where necessary.

**Figure 1:**
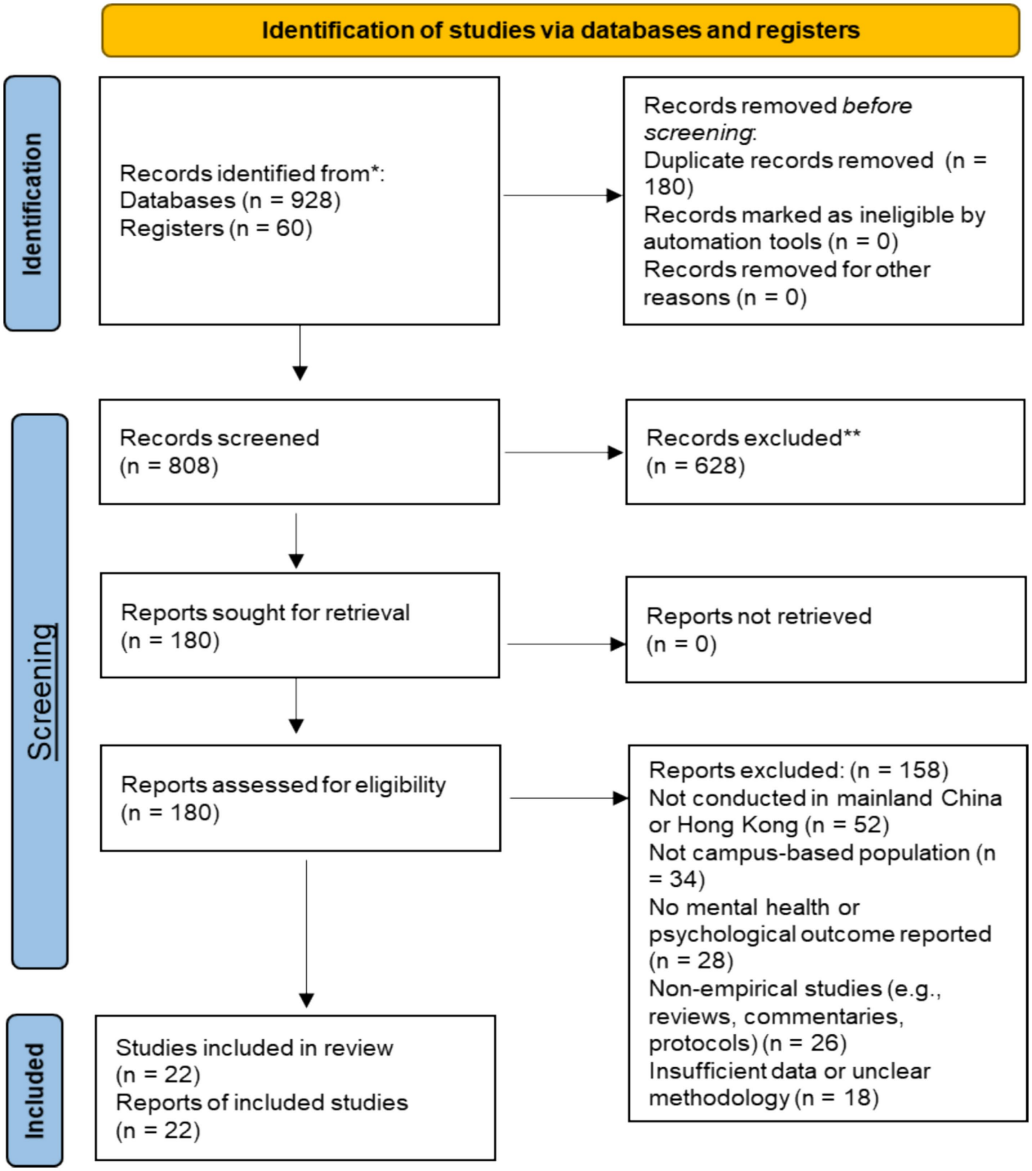
PRISMA flow diagram.

The entire study selection process is presented in a PRISMA 2020 flow diagram (Figure 1).

### 2.6 Data collection process

A standardized data abstraction form was used to extract data in this review. The data in each study were extracted by two reviewers to achieve accuracy and consistency.

The extracted data were cross-examined, and gaps were resolved through consensus and discussion. In cases of disagreement, the third reviewer was approached.

Some basic data-cleaning operations, including standardization of variable labels or formatting, were performed to ensure consistency between the studies.

### 2.7 Data items

Each included study provided the following data, which were extracted: author(s), year of publication, country of study, study design, and sample size. The exposure of the green space was also observed both objective (e.g., NDVI, GS-based indices, spatial buffers) and subjective measurements (e.g., perception of greenness, accessibility, quality, and usage of green spaces).

The outcome owing to the psychological state, both negative (e.g., stress, anxiety, depression, and psychological distress) and positive (e.g., life satisfaction, emotional well-being, happiness, and psychological restoration), was retrieved with respect to the predefined operational definition of mental condition, as well as the measurement instruments applied.

Other data involved analytical procedures, theoretical models (e.g., Attention Restoration Theory, Stress Reduction Theory), and significant results, such as the purpose and the strength of associations provided.

When specific mental health instruments were not explicitly reported in the original studies, they were recorded as “not reported” in the data extraction.

### 2.8 Quality appraisal

The quality of the included studies in terms of methodology was measured using the Mixed Methods Appraisal Tool (2018), which enables the qualitative, quantitative, and mixed-methods studies.

All studies were reviewed using criteria applicable to the study design, such as the suitability of the design, sufficiency of data collection procedures, the consideration of confounding factors, and the suitability of the outcome measurements.

Two reviewers also took part in the quality appraisal to help ensure consistency and reduce bias. Any discrepancies were to be resolved through discussion and agreement; in the event of a discrepancy, a third reviewer will be consulted.

### 2.9 Synthesis methods

Due to substantial heterogeneity across the included studies, a quantitative meta-analysis of effect sizes was not performed. Heterogeneity was observed in several key domains, including study design (cross-sectional, longitudinal, and experimental), green space exposure assessment (e.g., NDVI, GIS-based measures, perceived greenness), mental health outcome measures (e.g., PSS, PHQ-9, SWLS), and statistical reporting formats.

Although some studies reported effect sizes, the variability in measurement scales, analytical models, and reporting formats limited the feasibility of harmonizing these estimates for quantitative synthesis. In particular, differences in outcome operationalization and the absence of consistently reported standardized effect measures prevented meaningful aggregation.

Where partial comparability existed, the direction of associations (positive, null, or mixed) was extracted and categorized rather than quantitatively pooled. Therefore, a structured narrative synthesis approach was adopted following the guidance of Popay et al. (2006). Studies were grouped based on key characteristics, including measurement type (objective vs. subjective), study design, and outcome domain, and patterns in the direction and consistency of associations were identified across groups.

Mediating and moderating variables were classified based on the analytical approaches reported in the included studies. Mediation was defined as the testing of variables using formal statistical methods, such as indirect effect estimation, structural equation modeling, or bootstrapped mediation analysis. Moderation was defined as the use of interaction terms or subgroup analyses assessing differential effects across populations or contexts.

In cases where studies did not explicitly conduct formal mediation or moderation analyses, classification was based on the authors’ interpretations and theoretical framing of the relationships. This approach is acknowledged as a limitation, as it may introduce subjectivity in the categorization of mechanisms.

Effect sizes were not consistently reported across studies and, therefore, were not synthesized quantitatively; instead, the direction of associations was used.

## 3 Results

### 3.1 Study selection

The database search returned 928 records, and an additional 60 were found in Google Scholar, for a total of 988 records. After the removal of 180 duplicate records, 808 records were left to be screened.

Two reviewers screened titles and abstracts of these records to identify eligible studies. Of these, 628 records were excluded for irrelevance to the aims of the study.

The remaining 180 articles were listed for retrieval for full-text evaluation. Two independent reviewers were allowed to screen the full texts against prearranged inclusion and exclusion criteria, and any disagreements were to be resolved through discussion and, if necessary, consultation with a third reviewer.

Among all the 180 full-text articles evaluated, 158 articles were excluded due to the following reasons: not conducted in mainland China or Hong Kong (*n* = 52), not campus-based populations (*n* = 34), lack of mental health outcome (*n* = 28), and lack of sufficient data or inadequate methodology (*n* = 18).

A total of 22 studies met all inclusion criteria and were included in the final synthesis. The study selection process is illustrated in the PRISMA 2020 flow diagram (Figure 1).

### 3.2 Study characteristics

All included studies were carried out in mainland China and Hong Kong. International studies identified during the preliminary search were excluded from the eligibility assessment to ensure homogeneity with the stipulated geographical scope of this review.

Although the search included articles as early as 2000, publications dated 2020 to 2024 were included in the study as they met the inclusion criteria. This indicates a comparatively recent consideration of studies on campus green spaces and the mental health of students as a context.

The study designs were surveys, cross-sectional (*n* = 17), longitudinal (*n* = 3), and experimental or quasi-experimental (*n* = 2). The sample size ranged from 243 to 10,022.

The majority of studies employed cross-sectional designs (*n* = 17), followed by longitudinal (*n* = 3) and experimental or quasi-experimental designs (*n* = 2).

### 3.3 Green space exposure measures

The study of green space exposure was carried out in the context of a number of methodological concepts:

- Normalized Difference Vegetation Index (NDVI) was measured at 300–1500 m spatial buffers.
- Perceived greenness was measured through four metrics, which included visibility, access, quality, and utilization.
- The campus landscape typologies include green spaces, blue spaces, gray spaces, living spaces, and study spaces.
- The analysis of yoga practices during lockdown periods showed how outdoor spaces were used throughout the COVID-19 lockdowns.
- The study employed specific site exposure assessments, which focused on particular locations

Green space exposure was assessed using a range of methodological approaches. Objective measures included NDVI, GIS-based mapping, and spatial buffer analyses, while subjective measures captured perceived accessibility, quality, and use of green spaces. Fewer studies employed mixed approaches combining both objective and subjective indicators. Visual exposure assessments, such as street-view imagery, were also used in a limited number of studies.

### 3.4 Mental health outcomes assessed

The included studies assessed a range of mental health outcomes encompassing both negative psychological states and positive dimensions of well-being. Negative mental health indicators included depression, anxiety, and stress, typically measured using validated instruments such as the CES-D, PHQ-9, DASS, GAD-7, and PSS.

Furthermore, some studies evaluated psychosomatic symptoms and general psychological distress. Positive outcomes included life satisfaction, happiness, and broader psychological well-being, commonly assessed using instruments such as the Satisfaction With Life Scale (SWLS) and the Warwick–Edinburgh Mental Well-being Scale (WEMWBS).

### 3.5 Summary of effects

Across the 22 included studies:

- About 81.8% (18 studies) of the studies found statistically significant results between exposure to green spaces on the campus and mental health outcomes.
- Objectively measured greenness (e.g., NDVI) showed mixed and less consistent associations with mental health outcomes, with variability depending on spatial scale and analytical approach.
- Research examining campus landscape typologies indicated that restorative benefits varied across different types of green spaces, suggesting that not all green environments provide equal psychological benefits.
- Context-specific studies, including those conducted during COVID-19 restrictions, highlighted that the pattern of green space use and accessibility influenced the relationship between environmental exposure and psychosocial health outcomes.
- Overall, contact with green spaces on campus was identified as being related to reduced levels of psychological distress and increased levels of psychological well-being throughout the investigated studies. However, no direct comparison was possible due to varying study designs and measures.

Further examination of the included studies suggests that variability in reported associations is partly attributable to the method used to assess green space exposure, with subjective measures yielding more consistent positive findings compared to objective measures such as NDVI. Articles that used subjective variables to measure the quality of green space (i.e., perceived quality, accessibility, and applications) consistently reported positive relationships with mental health outcomes. Conversely, studies that used objective measures such as NDVI showed more inconsistent results, with some yielding significant results and others showing no statistically significant results. This suggests that perceived and experiential aspects of green space may play a more central role in influencing psychological outcomes than purely objective measures (Table 1).

**Table 1:** Characteristics of included studies.

Study ID	Author (Year)	Location	Study design	Sample size	Green space measure	Mental health outcomes	Measurement type	Self-reported scale (not reported)
S1	Liu et al. (2022)	China	Cross-sectional	2,800	Campus green spaces	Mental health, academic performance	Subjective	Self-reported scale (Not reported)
S2	Wang et al. (2017)	China	Cross-sectional	1,500	Social media green exposure	Emotions, mental health	Mixed	Perceived Restorativeness Scale (PRS)
S3	Bu et al. (2024)	China	Cross-sectional	1,200	Campus common spaces	Psychological restoration	Subjective	Self-reported scale (Not reported)
S4	Li and Carter (2025)	China	Cross-sectional	1,100	Environmental quality	Mental health, satisfaction	Subjective	Warwick–Edinburgh Mental Well-being Scale (WEMWBS)
S5	Bai X. et al. (2024)	China	Cross-sectional	2000	Visible green spaces	Mental well-being	Objective	Generalized Anxiety Disorder scale (GAD-7)
S6	Tian et al. (2025)	China	Cross-sectional	1,300	Greenness exposure	Anxiety	Objective	Perceived Stress Scale (PSS) / PRS
S7	Chen et al. (2025)	China	Cross-sectional	1,540	Audio-visual perception	Stress, restoration	Mixed	Generalized Anxiety Disorder scale (GAD-7)
S8	Diao et al. (2024)	China	Cross-sectional	1,200	Perceived greenness	Anxiety	Subjective	Perceived Restorativeness Scale (PRS)
S9	Du et al. (2022)	China	Cross-sectional	980	Landscape typology	Restoration	Subjective	Satisfaction With Life Scale (SWLS)
S10	Jiang et al. (2025)	China	Cross-sectional	1,100	Green space use	Well-being	Subjective	Self-reported scale (Not reported)
S11	Lu et al. (2022)	China	Cross-sectional	1750	Campus green exposure	Well-being	Mixed	Patient Health Questionnaire (PHQ-9) + Perceived Stress Scale (PSS)
S12	Lu et al. (2024)	China	Longitudinal	5,200	NDVI exposure	Stress, depression	Objective	Perceived Restorativeness Scale (PRS)
S13	Nie et al. (2024)	China	Cross-sectional	1,300	Landscape types	Restoration	Subjective	Perceived Stress Scale (PSS)
S14	Lin et al. (2023)	China	Cross-sectional	1,050	Yard exposure	Stress	Objective	Not reported
S15	Chen K. et al. (2021)	China	Cross-sectional	1800	Green exposure mechanisms	Mental health	Mixed	Perceived Stress Scale (PSS)
S16	Zhai et al. (2018a)	China	Cross-sectional	1,600	Environmental exposure	Stress	Objective	Self-reported scale (Not reported)
S17	Lai and Ma (2016b)	Hong Kong	Cross-sectional	1,200	Social-environmental factors	Well-being	Subjective	Ryff’s Psychological Well-being Scale
S18	Gao T. and McLellan (2018)	China	Cross-sectional	900	Psychological indicators	Well-being	Subjective	Not reported
S19	Wang et al. (2014)	China	Cross-sectional	2,100	Green exposure	Mental health	Objective	Not reported
S20	Zhang et al. (2020)	China	Cross-sectional	1,400	Urban greenness	Mental health	Objective	Perceived Restorativeness Scale (PRS)
S21	Huang et al. (2025)	China	Cross-sectional	52	Landscape perception	Psychological restoration	Subjective	Perceived Restorativeness Scale (PRS)
S22	Gao et al. (2025)	China	Cross-sectional	1,500	Perceived campus landscape characteristics	Psychological restoration / mental well-being	Subjective	Self-reported scale (Not reported)

Table 2 presents a synthesis of findings across the included studies, including the direction of associations, measurement approaches, and identified mediating or moderating factors.

**Table 2:** Synthesis of findings.

Study ID	Measurement type	Association direction	Key findings	Mediator/moderator	Quality rating
S1	Subjective	Positive	Higher perceived campus greenness linked to improved mental health and academic performance	Physical activity	Moderate
S2	Mixed	Positive	Social media green exposure is associated with better emotional outcomes	Social interaction	Moderate
S3	Subjective	Positive	Campus common spaces enhance psychological restoration	None reported	Moderate
S4	Subjective	Positive	Environmental quality is positively associated with mental health and satisfaction	None reported	Moderate
S5	Objective	Positive	Visible green spaces linked to improved well-being	None reported	Moderate
S6	Objective	Mixed	Greenness exposure is inconsistently associated with anxiety	None reported	Moderate
S7	Mixed	Positive	Audio-visual green perception reduces stress and enhances restoration	Perceptual factors	Moderate
S8	Subjective	Positive	Perceived greenness reduces anxiety	None reported	Moderate
S9	Subjective	Positive	Landscape typology influences restoration outcomes	None reported	Moderate
S10	Subjective	Positive	Frequency of green space use improves well-being	Usage behavior	Moderate
S11	Mixed	Positive	Campus green exposure is associated with well-being	None reported	Moderate
S12	Objective	Mixed	NDVI exposure shows variable effects on stress and depression	None reported	Moderate
S13	Subjective	Positive	Campus landscape types significantly affect restoration	None reported	Moderate
S14	Objective	Mixed	Yard-level greenness shows a weak association with stress	None reported	Moderate
S15	Mixed	Positive	Mechanisms of green exposure linked to improved mental health	Physical activity, social cohesion	Moderate
S16	Objective	Mixed	Environmental exposure is inconsistently linked to stress	None reported	Moderate
S17	Subjective	Positive	Social-environmental factors enhance well-being	Social cohesion	Moderate
S18	Subjective	Positive	Psychological indicators improve with green exposure	None reported	Moderate
S19	Objective	Mixed	Objective green exposure shows inconsistent mental health outcomes	None reported	Moderate
S20	Objective	Mixed	Urban greenness exhibits weak or null associations	None reported	Moderate
S21	Subjective	Positive	Landscape perception enhances restoration	None reported	Moderate
S22	Subjective	Positive	Perceived naturalness improves restoration and health	Usage patterns	Moderate

The majority of studies (18/22) reported statistically significant positive associations, although these findings were predominantly derived from cross-sectional designs and self-reported measures (Table 2).

Quality ratings and risk-of-bias assessments are based on study design, measurement validity, and analytical rigor, in line with the review objectives.

### 3.6 Mediating and moderating factors

The included studies identified several potential mechanisms linking campus green space exposure to mental well-being, including physical activity, social interaction, and place attachment.

Formal mediation analysis was explicitly conducted in a limited number of studies. For example, Diao et al. (2024) applied statistical mediation models to examine indirect effects of perceived green space on anxiety through factors such as frequency of use and nature connectedness. In such cases, mediation classification was based on established analytical techniques, including indirect effect estimation.

Conversely, several studies (e.g., Nie et al., 2024; Chen Y. et al., 2021) discussed potential mechanisms such as restoration or social cohesion without conducting formal mediation tests. These were classified as interpretative mediators, based on theoretical framing rather than statistical verification.

Moderation effects were less frequently examined. Where reported, moderation was identified based on subgroup analyses or interaction effects (e.g., differences by gender, usage patterns, or environmental context). However, the majority of studies did not formally test moderation, and such interpretations were therefore treated with caution.

Overall, evidence for mediation and moderation remains limited and heterogeneous, reflecting the predominance of cross-sectional designs and the inconsistent application of advanced analytical methods.

### 3.7 Study quality assessment

The methodological quality of included studies was assessed using the Mixed Methods Appraisal Tool (MMAT). Overall, most studies demonstrated moderate to high methodological quality.

The overall quality assessment indicated that most studies were of moderate to high methodological quality.

### 3.8 Comparative synthesis of findings

The comparative analysis of the studies showed that different types of green space measurements, study designs, and mental well-being measures yielded varying strengths and levels of consistency.

A tendency was observed for studies using subjective measures of green space exposure to report more consistently positive associations with mental health outcomes, whereas studies using objective measures such as NDVI showed more variable or mixed results (Table 2).

This pattern should be interpreted with caution, as the evidence is derived primarily from cross-sectional studies and heterogeneous measurement approaches.

Furthermore, studies focusing on psychological restoration and general well-being reported more consistent positive associations than those examining clinical outcomes such as depression and anxiety. Studies focusing on psychological restoration and general well-being reported more consistent positive associations, whereas studies examining clinical outcomes such as depression and anxiety showed more variable findings.

Across study designs, cross-sectional studies consistently reported positive associations; however, longitudinal and experimental studies provided stronger inferential evidence and demonstrated lower risk of bias, thereby offering more robust support for the relationship between campus green space exposure and mental well-being.

While patterns in the direction of associations and potential mechanisms were identified, the strength of evidence for these findings is limited by the predominance of cross-sectional designs and the inconsistent use of formal mediation and moderation analyses across studies.

## 4 Discussion

This systematic review examined the effects of exposure to campus green spaces on the mental health of university students in mainland China and Hong Kong. 22 relevant studies were identified for this review. Although the methodologies were varied across different geographical locations, there remains a consistent association between exposure to campus green spaces (e.g., parks and/ or other natural environments) and improved mental health, in keeping with results reported from previous studies that examined the benefits of restorative environments.

All studies included in this review consistently report an association between campus green space use and lower levels of depression, anxiety, stress, and psychological distress in general. However, the fact that the associations across studies were found to be consistently strong should warrant some caution in the interpretation of these results.

Although the majority of included studies employed cross-sectional designs, which limits causal inference, the presence of longitudinal and experimental studies in the evidence base provides stronger support for temporal associations between green space exposure and mental well-being. These studies strengthen the interpretation of the relationship; however, given their limited number, conclusions regarding causality should still be interpreted with caution.

Research has suggested possible mechanisms through which exposure to green space may be linked to improved psychological health, including psychological restoration; increased opportunities for social interaction; and increased levels of physical activity. For example, Diao et al. (2024) identified mediating effects of campus perception, frequency of use, and connection to nature on anxiety outcomes among subjects in their study, whereas Nie et al. (2024) provided evidence that benefits of psychological restoration vary according to certain attributes of the landscape (aesthetic complexity, shade, and environmental tranquility). These findings are also consistent with theoretical frameworks such as ART and SRT, which proposed that metacognitive and emotional pathways through which exposure to natural environments impact an individual’s sense of well-being exist. Nonetheless, these mechanisms should be interpreted as being associative, not causal.

Several examples of Green Space measurement are provided using the Normalized Difference Vegetation Index (NDVI). These provide a standardized, large-scale assessment of the amount of green space available to an individual but do not capture the subjective experience or perceptions of that green space. Conversely, the subjective measures of the distance from the individual, condition of the green space, and the ability to use the green space were consistently able to predict mental health outcomes for individuals who had used these methods to evaluate their green space exposure and thus, provide an indication that individual perception may be influencing psychological health more closely than the objective measures currently available. Therefore, it is critical to incorporate both objective and subjective measures in future studies to better understand the multifaceted nature of exposure to green space.

Contextual variability, in addition to measurement variability, was identified as an influence on the association of green space exposure and mental health outcomes. Studies conducted during the global pandemic revealed that there was a strong relationship between the use of green space during the COVID-19 lockdown and the well-being of individuals using green space outside of their residence. The research by Lin et al. (2023) illustrates that individuals who were exposed to their immediate green environments for short periods (i.e., while walking) experienced lower levels of stress, whereas those with longer exposure times were likely to be experiencing higher levels of pre-existing psychological distress. This indicates that green space exposure functions in varying capacities (i.e., coping and buffering) depending on contextual and individual factors.

Differences in student subgroups further emphasize the importance of equity and access in how green space is related to mental well-being. For example, there are differences in how gender, socioeconomic status, and where a student lives affect the relationship between green space and mental well-being; these findings indicate that different student groups do not derive equal benefits from available green infrastructure. In particular, students with limited access to natural settings off campus will rely more heavily on campus-based green spaces as their only source of nature; therefore, how and where green resources are provided on campus will be very important to those students.

While the observed associations are consistent, there are multiple methodological limitations in the evidence base supporting those associations. First, the majority of studies employed a cross-sectional design, which limits causal inference and increases the risk of reverse causality and unmeasured confounding. Second, there is significant variability in how green space is operationalized, including the use of different buffer sizes for NDVI, different scales for perceived greenness, and different types of landscapes. Third, there are very few studies that used objective behavioral or physiological measures in addition to subjective measures, and few studies used longitudinal or experimental designs that could establish temporal relationships.

In conclusion, longitudinal and quasi-experimental studies in this review provide relatively strong (though still limited) evidence of the temporal association between green space exposure and mental health. There is a need for further longitudinal, experimental, and multilevel studies using rigorous designs to establish causal pathways and to clarify mechanisms.

Based on the overall convergence of findings from different types of study designs, measurement techniques, and settings, green space exposure has been shown to have positive effects on the mental health of university students.

The findings reinforce the importance of developing accessible green areas on campus and providing access to these spaces in academic settings that experience higher levels of stress. Developing green spaces that incorporate design features that enhance usability or increase attractiveness (e.g., adding shade to seating, increasing the diversity of the overall setting, and having areas that promote rejuvenation) will promote the quality of green environments and their positive interactions.

### 4.1 Limitations

This review is limited by the predominance of cross-sectional studies, heterogeneous greenness metrics, and limited mechanistic testing. This review is subject to potential language bias, as only English-language studies were included despite the regional focus on mainland China and Hong Kong. Although databases indexing Chinese-language literature were searched, relevant non-English studies may have been excluded due to resource constraints, potentially limiting the completeness of the evidence base.

### 4.2 Implications for practice and policy

Universities should prioritize integrating accessible, high-quality green spaces into campus planning. Evidence suggests that these environments serve as scalable, cost-effective interventions for improving psychological well-being.

### 4.3 Recommendations

According to the evidence synthesized in the present review, strategic integration of green spaces should be considered as the priority of universities and policymakers as an element of a broader program of mental health and campus-well-being programs. The results highlight that green spaces are not only marked by ornamental features but also by infrastructural facilities that help minimize stress, boost emotional resilience, and facilitate everyday renewal among students. The institutions should thus increase the amount of accessible, high-quality green spaces, especially in high-pressure academic places like libraries, laboratories, and lectures, and also make them safe, well-maintained, and aesthetically appealing. The restorative experience can be significantly augmented by judicious landscape design to improve perceived greenness, provide shade, quality seating, water, and elements that promote biodiversity. Also, encouraging organized and unstructured nature-based interventions, e.g., outdoor study areas, walks, and green exercise, can increase the rate of student interaction with nature-based settings and reinforce mediating mechanisms, e.g., social cohesion and physical activity.

Future research must go beyond cross-sectional designs, which are widely used, by adopting longitudinal or experimental designs that can determine causal relationships between exposure to green spaces and mental health outcomes. The standardization of greenness measures such as NDVI buffer sizes, perceived greenness scales, and campus landscape typologies should be a priority for researchers to enhance comparability across studies. Further research is also necessary to study mediating factors, such as attentional restoration, emotional regulation, and social interaction, and moderating factors, such as gender, socioeconomic status, and residential context. Mechanistic knowledge can be enhanced through physiological measures, digital behavioral monitoring, and multimodal sensory measurements. Finally, since contact with nature is unevenly distributed across campuses and urban areas, universities and planners ought to instill equity-driven concepts in the green-space policies so that mental health benefits are available to all students.

## 5 Conclusion

This systematic review contains empirical indication that the use of and access to green spaces has a statistically significant positive impact on the mental health of university students and all adolescents, regardless of how the measurement was obtained (objective-using NDVI; or subjective-using perceived quality and utilization). The 22 studies found that use of and exposure to green spaces were positively correlated with reductions in depression, anxiety, stress, and primary psychosocial factor load and psychosomatic effects. While improvements in general mental health and perceived restoration occurred. These results are consistent with established theories of environmental psychology, specifically Attention Restoration Theory and Stress Reduction Theory, which each support the concept that natural environments offer visual and experiential opportunities for psychological rest.

The review also highlights the importance of the contextual and variable factors in moderating the effects of exposure to green spaces. The extent to which green spaces affected mental health was influenced by campus-specific scenery, sensory experience, closeness to functional academic areas, and the constraints imposed during the pandemic. Aesthetic, social, and experiential aspects of well-being were often more strongly related to subjective perceptions of greenness and objective measures of greenness. However, the domain is also limited by an excess of cross-sectional studies, heterogeneous measures of greenness, and unequal psychometric analyses, which hinder the identification of causal effects and underperform in comparative reliability of studies.

Despite these methodological shortcomings, the aggregate findings confirm the deliberate design and thoughtful care in maintaining green environments as a scalable, low-cost intervention to reinforce students’ mental well-being. Since universities face increasing levels of psychological stress among their students, green infrastructure can no longer be considered merely an aesthetic convenience but rather a part of mental health promotion and a long-term campus planning instrument.

## Data Availability

The raw data supporting the conclusions of this article will be made available by the authors, without undue reservation.
