# Supplementary material for: A systematic review of the Nexus between campus green spaces and mental well-being in mainland China and Hong Kong
Source: Front Psychol. 2026 Jun 19;17:1846925. doi: 10.3389/fpsyg.2026.1846925 (PMC13328092; doi:10.3389/fpsyg.2026.1846925)
Supplement: Supplementary file 1 [file Data_Sheet_1.docx]

Supplementary Material 1: Search Strategy

**Pubmed / MEDLINE**

(("green space"[Title/Abstract] OR greenness[Title/Abstract] OR "green infrastructure"[Title/Abstract] OR "natural environment"[Title/Abstract] OR NDVI[Title/Abstract])
AND
("university"[Title/Abstract] OR "college"[Title/Abstract] OR "campus"[Title/Abstract])
AND
("mental health"[Title/Abstract] OR "well-being"[Title/Abstract] OR stress[Title/Abstract] OR anxiety[Title/Abstract] OR depression[Title/Abstract]))

**Web of Science**

Uses TS = Topic Search

(("green space"[Title/Abstract] OR greenness[Title/Abstract] OR "green infrastructure"[Title/Abstract] OR "natural environment"[Title/Abstract] OR NDVI[Title/Abstract])
AND
("university"[Title/Abstract] OR "college"[Title/Abstract] OR "campus"[Title/Abstract])
AND
("mental health"[Title/Abstract] OR "well-being"[Title/Abstract] OR stress[Title/Abstract] OR anxiety[Title/Abstract] OR depression[Title/Abstract]))

**CNKI (China National Knowledge Infrastructure)**

Use Chinese + English mix

("校园 绿地" OR "绿色空间" OR "绿化" OR "自然环境")
AND
("大学生" OR "高校学生" OR "校园")
AND
("心理健康" OR "心理幸福感" OR "压力" OR "焦虑" OR "抑郁")

**CrossRef**

Keyword-based search

("green space" OR greenness OR "natural environment" OR NDVI)
AND
("university students" OR "college students" OR campus)
AND
("mental health" OR well-being OR stress OR anxiety OR depression)

**OpenAlex**

("green space" OR greenness OR "green infrastructure" OR "natural environment")
AND
("university students" OR "college students" OR campus)
AND
("mental health" OR "psychological well-being" OR stress OR anxiety OR depression)

**Google Scholar**

("campus green space" OR greenness)
AND
("university students" OR "college students")
AND
("mental health" OR well-being OR stress OR anxiety OR depression)
